# Update on pathology laboratory development and research in advancing regional cancer care in Malawi

**DOI:** 10.3389/fmed.2024.1336861

**Published:** 2024-01-16

**Authors:** Amy J. Brownlee, Morgan Dewey, Maganizo B. Chagomerana, Tamiwe Tomoka, Maurice Mulenga, Shiraz Khan, Coxcilly Kampani, Fred Chimzimu, Julie M. Gastier-Foster, Kate D. Westmoreland, Nmazuo W. Ozuah, Robert Krysiak, Chikondi Malamba-Banda, Matthew S. Painschab, Satish Gopal, Yuri Fedoriw

**Affiliations:** ^1^Department of Pathology and Laboratory Medicine, School of Medicine, University of North Carolina, Chapel Hill, NC, United States; ^2^School of Medicine, University of Colorado Anschutz Medical Campus, Aurora, CO, United States; ^3^University of North Carolina (UNC) Project-Malawi, Lilongwe, Malawi; ^4^Division of Infectious Diseases, School of Medicine, University of North Carolina, Chapel Hill, NC, United States; ^5^Kamuzu Central Hospital, Malawi Ministry of Health, Lilongwe, Malawi; ^6^Departments of Pediatrics and Pathology & Immunology, Baylor College of Medicine, Texas Children's Hospital, Houston, TX, United States; ^7^Division of Pediatric Hematology Oncology, Department of Medicine, University of North Carolina, Chapel Hill, NC, United States; ^8^Lineberger Comprehensive Cancer Center, School of Medicine, University of North Carolina, Chapel Hill, NC, United States; ^9^Section of Hematology and Oncology, Department of Pediatrics, Baylor College of Medicine, Houston, TX, United States; ^10^Malawi University of Science and Technology, Limbe, Malawi; ^11^Malawi-Liverpool-Wellcome Trust Clinical Research Program, Blantyre, Malawi; ^12^Division of Hematology, Department of Medicine, University of North Carolina, Chapel Hill, NC, United States; ^13^Center for Global Health, National Cancer Institute (NIH), Bethesda, MD, United States

**Keywords:** Malawi, pathology, pathology laboratory development, cancer research, cancer care, Africa, global health

## Abstract

The pathology laboratory at Kamuzu Central Hospital (KCH) in Lilongwe, Malawi was established in 2011. We published our initial experiences in laboratory development and telepathology in 2013 and 2016, respectively. The purpose of this paper is to provide an update on our work by highlighting the positive role laboratory development has played in improving regional cancer care and research. In addition, we provide a summary of the adult pathology data from specimens received between July 1, 2011, and May 31, 2019, with an emphasis on malignant diagnoses. We compare these summaries to estimates of cancer incidence in this region to identify gaps and future needs.

## 1 Introduction

Pathology services are crucial to addressing the escalating cancer burden in Africa that has resulted from the human immunodeficiency virus (HIV) epidemic, demographic expansion and aging, and shifting risk factor profiles due to a broad adoption of a westernized lifestyle (1). By providing healthcare systems with accurate diagnoses, pathology laboratories facilitate disease recognition and monitoring at the individual and population levels and assist with clinical trial implementation and cancer research training. In these ways, pathology laboratories are invaluable in informing regionally appropriate treatment approaches and determining resource allocation to further develop the clinical and research infrastructure needed to optimize regional cancer care.

Prior to 2011, Lilongwe, the capital and most populous city of Malawi, had no local pathology services. Pathology specimens were sent to the United Kingdom (before 1981) or to Blantyre, Malawi to be reviewed by the sole pathologist in the country (1982–1995). As a result, immediate diagnostic information and thus timely and appropriate care of patients could not be provided. In addition, there was a large proportion of either misdiagnosed or undiagnosed cancer cases.

Through a longstanding collaboration between the Malawi Ministry of Health (MoH), Kamuzu Central Hospital (KCH), and the University of North Carolina at Chapel Hill (UNC), a pathology laboratory was opened in Lilongwe, Malawi, in 2011, representing the second pathology lab in a country of, at that time, approximately 16 million people. Here we present an update on laboratory development (2) and telepathology (3) with a special emphasis on the role this capacity development has played in regional cancer control. We also review the adult pathology data from KCH to determine how the case mix compares to regional cancer statistics. We hope to use this data to guide resource allocation, e.g., grant funding and research and training programs, needed for effective local cancer care.

## 2 Materials and methods

### 2.1 Ethics statement

The included research has been approved by the UNC institutional review board (IRB) and the Malawi National Health Sciences Research Committee. The IRB approval covers analysis of data collected through the UNC-Project Malawi cancer program.

### 2.2 Pathology data sources and analysis

This is a single center, retrospective case series of pathology specimens taken from adult patients aged 18 years or older and reviewed at the KCH pathology laboratory between July 1, 2011, and May 31, 2019. After specimen collection, a standardized pathology requisition form is completed by requesting clinicians and provided to the laboratory. This form includes basic patient information (age, date of birth, gender, and HIV status), brief clinical details, and specimen information (body site, tissue type, and date of collection). Once a specimen is received by the laboratory, it is assigned a unique case number, and details from the requisition form are recorded in a secure institutional database. Diagnostic conclusions are recorded in the same database after specimen processing and pathologic interpretation. Data from the KCH laboratory was initially stored using Microsoft Access until switching to the LABDAQ Laboratory Information System (LIS) in October 2018. The data pulled from the more recent LIS database was formatted to align with the Access formatting for ease of analysis.

We manually reviewed the database to categorize each specimen according to body system and diagnostic category. Diagnoses were categorized as malignant, premalignant, benign, non-neoplastic (infectious, reactive, or normal), non-diagnostic, or differential. The differential category was created for specimens that contained lesional tissue but could not be confidently categorized into one of the other defined categories. The body system and diagnostic category labels were later used to filter data to help generate descriptive statistics.

Descriptive statistics were used to summarize patient characteristics, specimen types, and final diagnoses. Log binomial regression was used to estimate HIV prevalence ratios (PR) and the corresponding 95% CI of the association between sex and prevalence of HIV for all participants and sex and prevalence of HIV among participants with malignant diagnoses. Bivariable and multivariable logistic regression were used to examine patient characteristics associated with malignant diagnosis. All analyses were conducted using Stata version 14.2 (StataCorp, College Station, Texas USA). A two-sided alpha value of 0.05 was used to assess statistical significance.

## 3 Results

### 3.1 Laboratory development and diagnostic procedures

As previously described (2), the KCH laboratory became operational in July 2011 and was staffed by one full-time Malawian pathologist who reviewed 95% of all specimens and was supported by international volunteers from Pathologists Overseas and weekly telepathology sessions with pathologists at UNC. The histology laboratory was staffed by three Malawian histology technicians. By July 2013, a handful of key immunohistochemical (IHC) stains (Ki-67, p16, LANA, CD3, CD20, CD45, ER, and PR) were implemented primarily to support the KCH Lymphoma Study, an observational clinical cohort of lymphoproliferative disorders (LPDs) that continues to enroll to this day (3).

**Figure 1:**
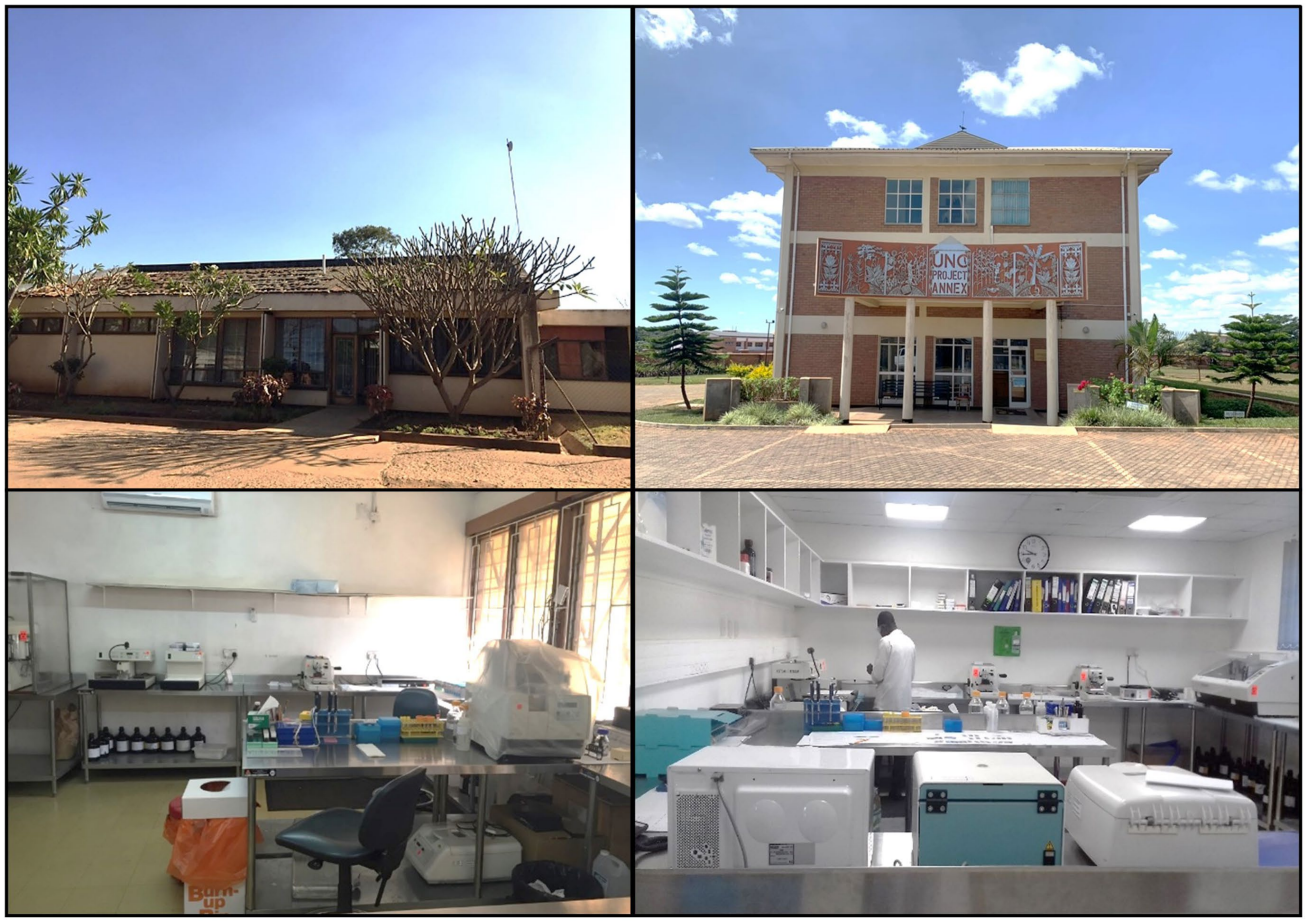
Pictures of the old (left panel) and new (right panel) pathology laboratory building (top panel) and indoor laboratory space (bottom panel).

Since 2013 the KCH laboratory has continued to grow (Table 1) and now includes three Malawian pathologists, five histology technicians, one cytotechnologist, and one administrative assistant. The pathologists all trained in South Africa and the other laboratory personnel received training either in South Africa or elsewhere in the region. The IHC panel has expanded to include markers used in the work-up of LPDs (BCL2, BCL6, CD10, CD30, CD138, MPO, MUM1 PAX5, and TdT), breast cancer (HER2), tumor differentiation (AE1/AE3, S100, synaptophysin, and neurofilament), and, with the support of Baylor College of Medicine Children’s Foundation-Malawi, pediatric small round blue cell tumors (CD99, desmin, and myogenin). Due to expanding diagnostic capacity, a new laboratory was built in 2017 growing the physical lab from 676 ft^2^ to 1,557 ft^2^ to accommodate more personnel and laboratory equipment (Figure 1). This ongoing development has been supported by continuous rounds of NIH funding along with continued and expanding US-based institutional support, and, most importantly, financial support from the Malawi Ministry of Health.

**Table 1:** Timeline of key pathology laboratory developments at Kamuzu Central Hospital (KCH).

Year	Progress
Before 1981	Specimens sent to St. Thomas’ Hospital in United Kingdom.
1982–1995	Specimens sent to Blantyre, Malawi: Queen Elizabeth Central Hospital (1982–95) and University of Malawi College of Medicine (1995–2011).
2010–2011	Acquisition of laboratory space at KCH in Lilongwe followed by renovations, equipment installation, and validation procedures.
2011	KCH laboratory operational in Lilongwe with one Malawian pathologist.
2013	Manual IHC and digital telepathology implemented. First clinical cancer study established (KCH Lymphoma Study).
2016	New Malawian pathologist. Breast cancer cohort initiated.
2017	New laboratory space with all new laboratory equipment opened.
2018	New Malawian pathologist. Updated laboratory information system (LIS).
2021	Established partnership with Baylor College of Medicine Children’s Foundation-Malawi resulting in expanded IHC menu. Obtained automated slide stainer and coverslipper.
2023	New Malawian pathologist.

As previously described, we continue to conduct weekly clinicopathologic telepathology conferences followed by secondary review at UNC for patients with LPDs, and have maintained remarkably high concordance between real-time and final diagnoses (3). In addition, as of 2021, we also conduct pediatric clinicopathology conferences with the support of Baylor College of Medicine Children’s Foundation-Malawi. Both conferences are attended by US and Malawian pathologists, Malawi-based US medical oncologists, Malawian medical officers, other Malawian clinical and support staff including nursing, and occasionally, US-based trainees.

### 3.2 Adult pathology data analysis

**Figure 2:**
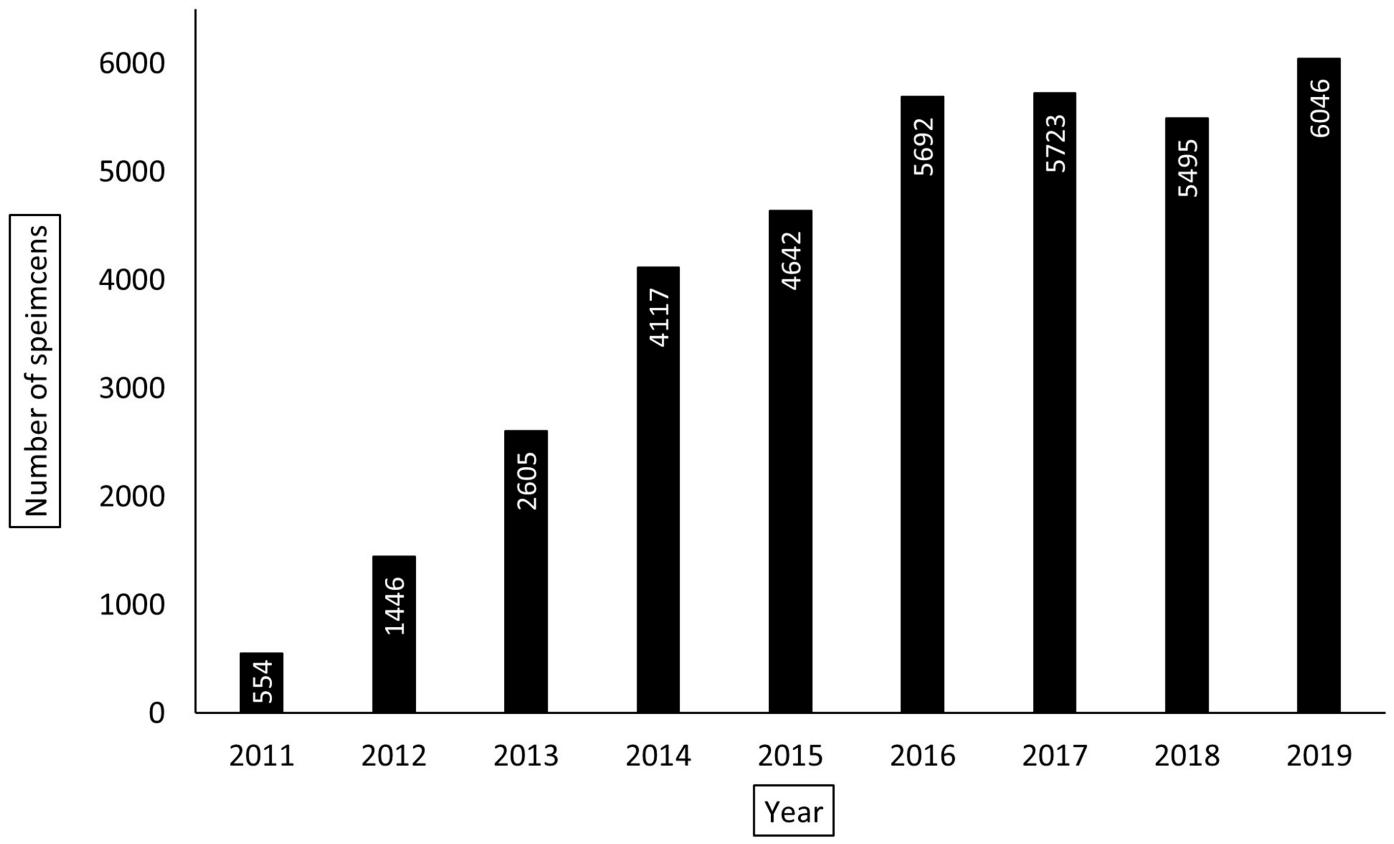
Yearly total number of adult pathology specimens processed by Kamuzu Central Hospital (KCH) pathology laboratory between July 1, 2011, and May 31, 2019. For the year 2019, the total number of specimens were extrapolated using the average number of specimens reviewed per month between January 1, 2019, and May 31, 2019. For the year 2011, only the total number of specimens received between July 1, 2011 and December 31, 2011 are reported.

The KCH pathology laboratory received 32,793 specimens from adult patients aged 18-years or older between July 1, 2011, and May 31, 2019. The total number of adult specimens reviewed by year is shown in Figure 2, growing from approximately 92 specimens per month in 2011 to approximately 504 specimens per month in 2019. Specimen and patient characteristics are shown in Tables 2, 3. Unfortunately, due to the nature of our dataset, the absolute number of unique individuals within this group could not be reliably determined. However, based on our prior experience (2) and the nature of the healthcare system in Malawi, we estimate that greater than 90% of specimens came from unique individuals. The reported patient characteristics are based on the data associated with each specimen received. Of all specimens received, 91% (*n* = 29,919) were diagnostic. Most were histology specimens (77%, *n* = 25,248), the plurality came from the female reproductive system (44%, *n* = 14,413), and the majority of diagnoses were non-malignant (57%, *n* = 18,585). Overall, mean age was 44 years. Gender was provided for the majority (98%, *n* = 32,076) of cases with 70% (*n* = 22,432) coming from female patients. HIV status was known for 51% (*n* = 16,858) of cases, and of the cases with known HIV status, 34% (*n* = 5,684) were HIV-positive.

**Table 2:** Specimen characteristics (*n* = 32,793).

Diagnosis category		
Malignant	9,629	29%
Premalignant	1,439	4%
Non-malignant	18,585	57%
Infectious	1,393	4%
Reactive	8,705	27%
Benign	3,836	12%
Normal	4,650	14%
Nondiagnostic	2,874	9%
Differential	267	1%
Specimen type		
Histology	25,248	77%
Cytology	7,532	23%
Autopsy	13	<0.1%

**Table 3:** Summary of patient characteristics provided on pathology requisition forms.

	Total	Malignant	Other
	*n* = 32,793	*n* = 9,629	*n* = 23,164
Age, mean (SD)	44 (16)	50 (16)	42 (1)
Gender, *n* (%)			
Provided	32,076 (98%)	9,422 (98%)	22,654 (98%)
Female	22,432 (70%)	5,695 (60%)	16,737 (74%)
Male	9,644 (30%)	3,727 (40%)	5,917 (26%)
Not provided	717 (2%)	207 (2%)	510 (2%)
HIV Status, *n* (%)			
Provided	16,858 (51%)	4,680 (49%)	12,178 (53%)
Positive	5,684 (34%)	1,927 (41%)	3,757 (31%)
Negative	11,174 (66%)	2,753 (59%)	8,421 (69%)
Not provided	15,935 (49%)	4,949 (51%)	10,986 (47%)

**Figure 3:**
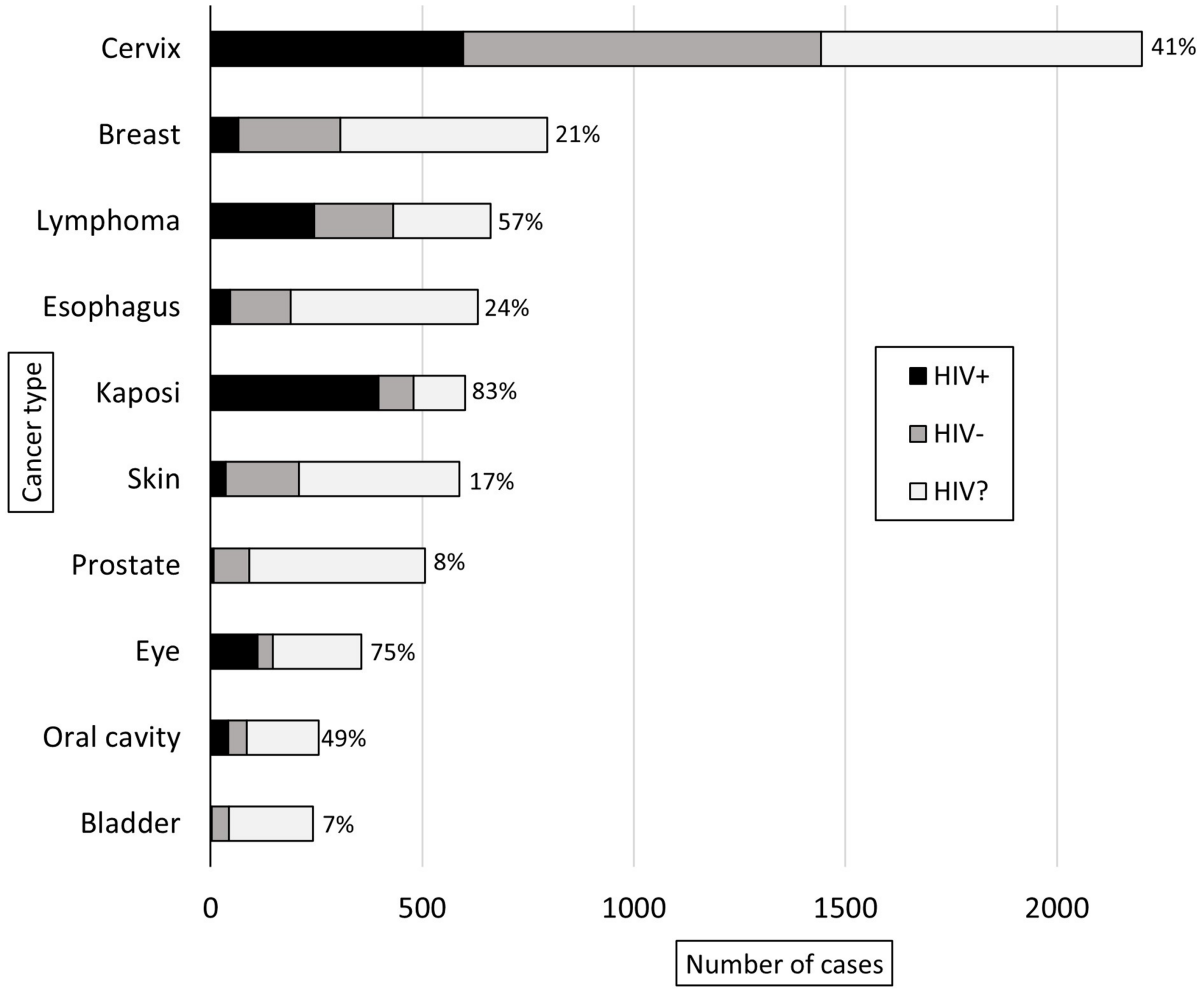
Ten most common adult malignant diagnoses rendered at KCH pathology laboratory between July 1, 2011, and May 31, 2019. The bars are divided to show the proportion of patients with reported HIV status (HIV-positive in black, HIV-negative in gray) and unknown HIV status (HIV?). For the patients with reported HIV status, the proportion of HIV-positive cases (%) is reported.

**Figure 4:**
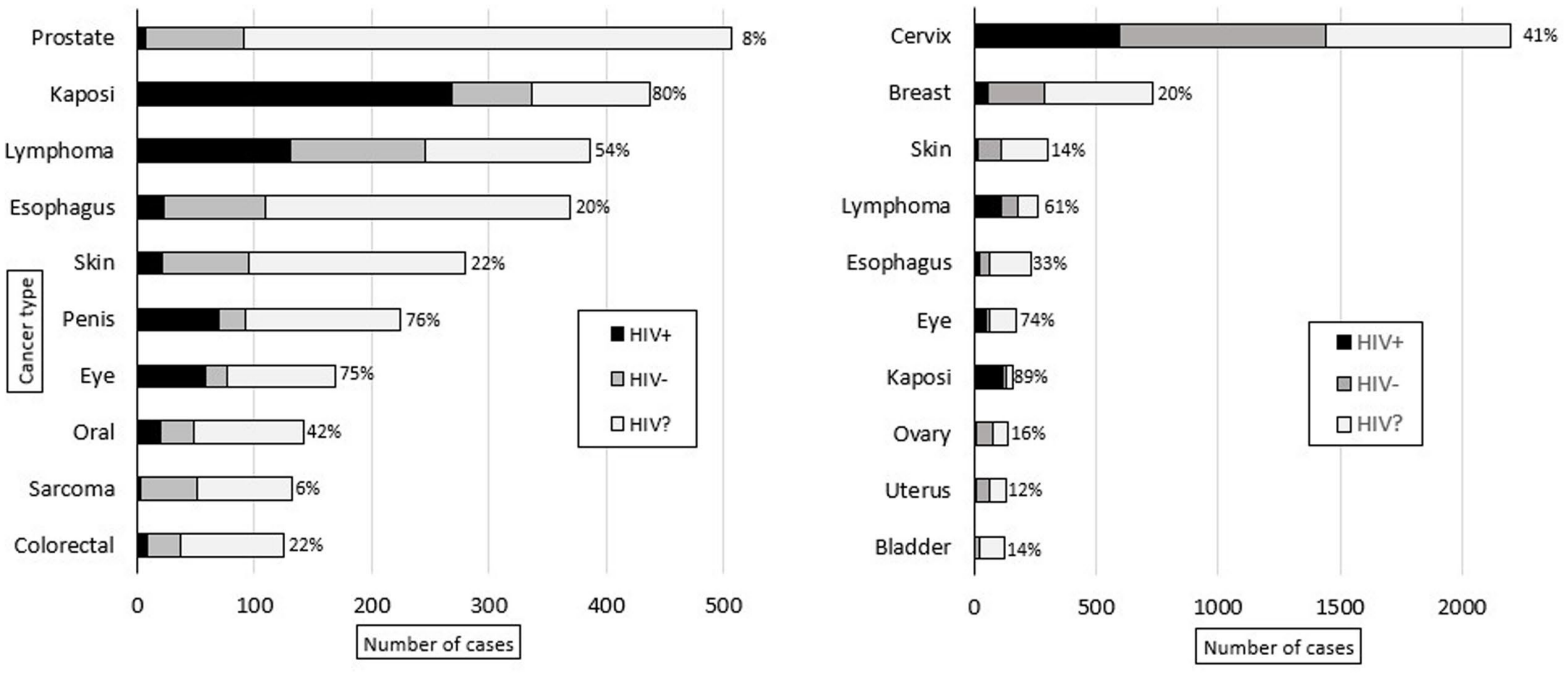
Ten most common adult male (left) and female (right) malignant diagnoses rendered at KCH pathology laboratory between July 1, 2011, and May 31, 2019. The bars are divided to show the proportion of patients with reported HIV status (HIV-positive in black, HIV-negative in gray) and unknown HIV status (HIV?). For the patients with reported HIV status, the proportion of HIV-positive cases (%) is reported.

Malignant diagnoses accounted for 29% (*n* = 9,629) of specimens, and HIV status was known for 49% (*n* = 4,680) of these cases. In malignant cases, compared to all other cases, patients were older (50 vs. 44 years, *p* < 0.001), had a higher prevalence of HIV infection (41% vs. 31%, *p* < 0.001), and were more commonly male (74% vs. 60%, *p* < 0.001). Figure 3 shows the ten most common malignant diagnoses with cervical cancer (23%, *n* = 2,201) being the most common. Based on organ system, the most common malignant diagnoses were in the female reproductive (28%, *n* = 2,736), gastrointestinal (15%, *n* = 1,449), dermatologic (11%, *n* = 1,066), and genitourinary (11%, *n* = 1,036) systems. The highest HIV prevalence was seen in Kaposi sarcoma (KS) (83%) and eye cancer (75%, predominantly primary squamous cell carcinoma), and the lowest HIV prevalence was seen in prostate (8%) and bladder (7%) cancer. Figure 4 shows the ten most common malignant diagnoses in men and women, respectively, along with HIV prevalence by cancer type.

**Figure 5:**
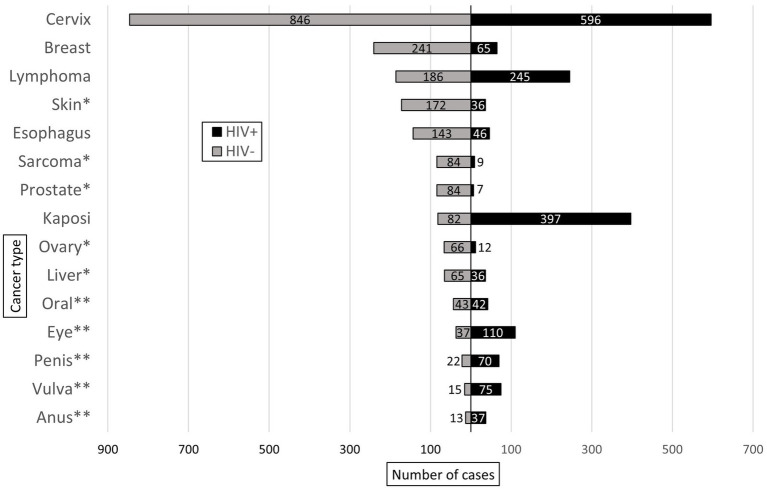
Ten most common malignant diagnoses rendered in HIV-positive (HIV+) and HIV-negative (HIV-) patients at KCH pathology laboratory between July 1, 2011, and May 31, 2019. * and ** indicate malignant diagnoses unique to the most common malignant diagnoses in the HIV- and HIV+ groups, respectively.

Of the malignant cases with known HIV status (*n* = 4,680), 41% (*n* = 1,927) were HIV-positive, and these had a higher proportion of malignant diagnoses (34%, *n* = 1,927) compared to HIV-negative cases (25%, *n* = 2,753, *p* < 0.001). Figure 5 shows the ten most common malignant diagnoses by HIV status. The most common malignancy in each group was cervical cancer, which accounted for 31% of both malignant HIV-negative (*n* = 846) and HIV-positive cases (*n* = 596). Overall, HIV prevalence was lower in cases with female gender (PR = 0.80; 95% CI: 0.80–0.88, *p* < 0.001), but there was no difference in HIV prevalence between male and female cases among malignant diagnoses (PR = 0.94; 95% CI: 0.87–10.1, *p* = 0.079).

Table 4 shows bivariable and multivariable associations between patient characteristics (age, gender, and HIV status) and a malignant diagnosis. A decade increase in age (adjusted odds ratio (aOR) = 1.42; 95% CI: 1.38–1.46) and HIV infection (aOR = 1.74; 95% CI: 1.61–1.87) were associated with increased odds of a malignant diagnosis while female gender (aOR = 0.51; 95% CI: 0.47–0.55) was associated with reduced odds of a malignant diagnosis.

**Table 4:** Association of patient characteristics with malignant diagnosis.

	Bivariable OR (95% CI)	Multivariable OR (95% CI)
Age, per decade	1.39 (1.37–1.41)	1.42 (1.38–1.46)
Female gender	0.54 (0.51–0.57)	0.51 (0.47–0.55)
HIV infection	1.57 (1.46–1.68)	1.74 (1.61–1.87)

OR, odds ratio; CI, confidence interval. *Bivariable analyses include only specimens with known patient age (*n* = 32,793), gender (*n* = 32,076), or HIV status (*n* = 16,858). **Multivariable analyses include only specimens with known patient age, gender, and HIV status (*n* = 16,487).

### 3.3 Research and clinical trials

**Figure 6:**
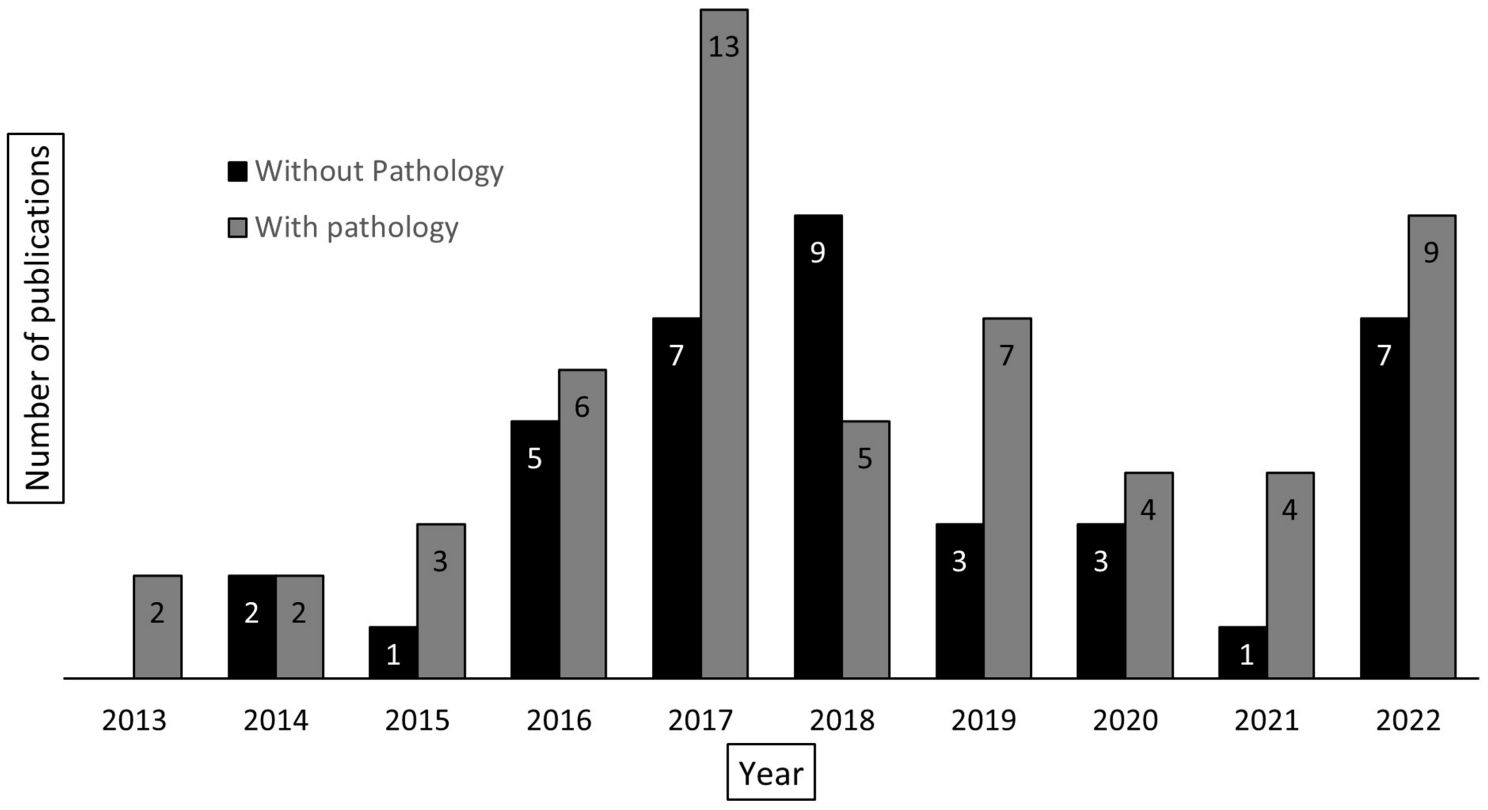
Total number of cancer-related publications, reported by year, associated with UNC-Project Malawi (*n* = 93). The columns are divided into those publications requiring pathology laboratory services (gray, *n* = 55) and those not requiring pathology laboratory services (black, *n* = 38).

Figure 6 shows the total number of cancer-related publications (*n* = 93) from UNC-Project Malawi cancer program between 2013 and 2022 in addition to publications specifically made feasible by pathology laboratory development and diagnostic services at KCH (*n* = 55, 59%). The majority (*n* = 30, 55%) of this latter group includes publications related to the study of LPDs. However, the KCH laboratory has also been involved in the research of esophageal cancer (4–6), breast cancer (7, 8), cervical cancer (9–11), melanoma (12), head and neck squamous cell carcinoma (13), and KS (14–19).

## 4 Discussion

Within a little over a decade, continual Malawi MoH investment in the KCH pathology laboratory along with efficient use of grant funding and ongoing research and training collaborations between the Malawi MoH, KCH, and UNC have greatly enhanced cancer care and research in Malawi.

For example, rapid diagnoses, robust clinical and follow-up data, and histologic confirmation of every case in the KCH lymphoma cohort gave the opportunity to advance understanding of the natural history, disease distribution, and unique biology of LPDs in this region. Firstly, it was recognized that most patients who were biopsied for cervical lymphadenopathy had malignant diagnoses (35%) rather than tuberculosis (6%), the latter of which was believed to be the most common cause of cervical lymphadenopathy in Africa at the time (20). Later, several case reports and case series expanded knowledge and recognition of rare subtypes of LPDs seen at KCH including extranodal NK/T-cell lymphoma (21), Rosai-Dorfman disease (22), primary effusion lymphoma (23), plasmablastic lymphoma (24), and multicentric Castleman disease (25–28). Through facilitating clinical trial enrollment, the KCH lymphoma study has also shown that CHOP, R-CHOP, and modified EPOCH are safe, effective, and feasible treatment regimens for aggressive non-Hodgkin lymphoma (NHL) (29), diffuse large B-cell lymphoma (DLBCL) (30), and other high-risk NHLs, e.g., plasmablastic lymphoma (31), respectively. Studies combining pathologic diagnoses with outcomes data have provided valuable information regarding prognosis and treatment feasibility in patients with DBLCL (32, 33), classic Hodgkin lymphom (34), acute lymphoblastic leukemia/lymphoma (35), Burkitt lymphoma (36–41), and relapsed or refractory lymphoma (42), and cost-effectiveness studies have further supported the feasibility of treating DLBCL in Malawi (43, 44). Lastly, gene expression profiling performed on pathologically confirmed cases of DLBCL provided one of the first molecular characterizations of DLBCL in Africa and showed that there was marked gene expression differences by HIV status in the KCH cohort and that prognostication systems used in resource-rich settings (e.g., cell of origin subtype) could not reliably predict outcomes (45). These findings suggest a tumor biology unique to this setting which has spurred additional investigations.

Due to the primary driving forces behind diagnostic laboratory development at KCH, there may be a differential representation of diseases in our cohort compared to population-based cancer registry statistics. For example, while lymphoma is estimated to be the seventh most common adult malignancy in Malawi representing 4% of total cancer cases in 2020 (46), it is over-represented in our cohort being the third most common malignancy and representing 7% of our cancer cases. Conversely, there is a possible under-representation of cervical (23% vs. 26%) and esophageal (7% vs. 11%) cancers in our cohort compared to cancer registry estimates (46). A major complicating factor in analyzing the data is limited population-based cancer registry data in Malawi and Africa in general (47). Additional research is needed to determine if and why specific cancers may be under-represented in the KCH database, and how best to address care and research gaps of these malignancies.

Due to the continued high prevalence of HIV infection in Malawi, with an adult prevalence rate of 7% reported in 2022 (48), HIV-associated malignancies are a prominent focus of research programs in this region and health care providers are likely very familiar with managing these diseases. Compared to our prior report in 2013 (2), a much higher proportion of malignant cases at KCH had known HIV status (49% vs. 11%), especially in cancers known to be associated with HIV infection: KS (80%), cervical cancer (66%), and lymphoma (65%). In our cohort, HIV was associated with increased risk of malignancy, the three AIDS-defining malignancies (cervical cancer, lymphoma, and KS) were in the top five most common cancer diagnoses, and HIV prevalence among all malignant and non-malignant cases with known HIV status was 34%. Clearly, HIV continues to impact cancer incidence in Malawi and HIV-infected individuals continue to have increased morbidity compared to the general population.

While a continued focus on HIV-associated diseases is essential, other variables should be considered in cancer control program development. With the scale up of antiretroviral treatment (ART) (49) and evolving risk factors such as population growth and aging (50) and adoption of westernized lifestyles, the neoplastic milieu is changing and clinical and research programs must also evolve. Our data again shows that increasing age is associated with malignancy; therefore, continued capacity development is needed to address the growing cancer burden in this aging population. Secondly, gastrointestinal cancers accounted for 15% of malignant diagnoses in our cohort, second only to female reproductive cancers (28%). Other than esophageal cancer, we do not have established projects focusing on other malignancies in this important group. Lastly, male gender was again associated with malignancy in bivariable and multivariable analysis with 39% of diagnoses made in male patients being malignant compared to 25% in female patients. This statistic may be misleading when one considers the impact of cervical cancer screening programs which result in a higher number of nonmalignant specimens among women compared with men. And, of note, regional statistics have consistently shown a higher cancer incidence in women than men (50, 51).

There are limitations to this work. As previously described (2), this data set comes from a national teaching hospital in a resource-limited setting, likely resulting in significant selection and referral bias compared to population-based cancer registries. KCH cancer patients tend to present with advanced disease after a long referral process. And, unfortunately, many of patients die before presenting for care. As previously discussed, malignancies that are a focus of active research programs will be overrepresented, and malignancies that may be difficult to diagnose pathologically, e.g., visceral sites or malignancies for which there are limited treatment options will be underrepresented. Next, demographic data is obtained from handwritten laboratory requisition forms filled out by busy clinicians at the time of specimen collection and transcribed into the laboratory information system (LIS). Due to typographical errors and absence of an electronic medical record (EMR), it is difficult to maintain complete and accurate records for patients seen at KCH. While KCH theoretically serves a referral population of 10 million from the Central and Northern Regions of Malawi, geospatial analyses are needed to determine exactly where patients are coming from, which regions are underserved, and why. In addition, an analysis of local referral patterns and reasons for diagnosis and treatment delays is needed. Even with these limitations, this data and continued efforts in laboratory development can help inform cancer control planning, especially in a region with evolving but still limited population-based cancer registries (47).

Despite the ongoing growth and success witnessed over the past decade, there are countless opportunities for further advancing self-sufficiency and optimizing regional and resource appropriate cancer care. A key focus will be to coordinate diagnostic capacity development with available treatment options and utilize our pathology data to support expansion of the cancer care infrastructure at KCH. Currently, traditional cytotoxic chemotherapy and endocrine therapies are routinely available and provided free of charge to patients by the Malawi MoH as part of universal health coverage. Therefore, treatment options for cancers that respond to these therapies, such as hematologic malignancies, are good. However, treatment options for solid tumors which often require radiation and surgical oncologic approaches are limited. Radiation is not yet available at KCH but is planned to be implemented in 2024. Surgical oncology services are available, but the capacity is limited as the demand for curative tumor resections far outstrips the supply of surgeons and operating room capacity. In addition, newer, more expensive therapies such as rituximab and trastuzumab are occasionally available to patients through the MoH, but if not, private access to similar therapies is often limited as the demand is low given the extremely high price of these therapies.

In conclusion, diagnostic pathology services are essential for advancing cancer care and research. Not only do they provide rapid diagnoses that impact treatment decisions for individual patients, but also, they provide tissue needed to study biology of disease and identify regionally appropriate and clinically actionable targets that can be explored through clinical trials. Research programs also provide an avenue for training local cancer researchers that can continue to advance cancer control efforts. With the compound burden of continued high prevalence of HIV-associated malignancies, a growing incidence of malignancies associated with population aging and westernized lifestyle, and a notable scarcity of diagnostic pathology services in this area, hospital-based cancer registries with robust clinical and demographic data paired with pathologically confirmed cancer diagnoses are invaluable for informing regional and resource appropriate cancer control program development.

## Data availability statement

The raw data supporting the conclusions of this article will be made available by the authors, without undue reservation.

## Ethics statement

The studies involving humans were approved by University of North Carolina Institutional Review Board Malawi National Health Sciences Research Committee. The studies were conducted in accordance with the local legislation and institutional requirements. Written informed consent for participation was not required from the participants or the participants’ legal guardians/next of kin in accordance with the national legislation and institutional requirements.

## Author contributions

AB: Conceptualization, Formal analysis, Methodology, Supervision, Writing – original draft, Writing – review & editing. MD: Data curation, Writing – review & editing. MC: Formal analysis, Methodology, Writing – review & editing. TT: Data curation, Writing – review & editing. MM: Data curation, Writing – review & editing. SK: Data curation, Writing – review & editing. CK: Data curation, Writing – review & editing. FC: Data curation, Writing – review & editing. JG-F: Funding acquisition, Resources, Writing – review & editing, Project administration. KW: Data curation, Writing – review & editing. NO: Resources, Writing – review & editing, Project administration. RK: Data curation, Methodology, Writing – review & editing. CM-B: Data curation, Methodology, Writing – review & editing. MP: Data curation, Methodology, Writing – review & editing. SG: Data curation, Methodology, Resources, Writing – review & editing. YF: Conceptualization, Data curation, Formal analysis, Funding acquisition, Methodology, Resources, Supervision, Writing – original draft, Writing – review & editing, Project administration.
